# 

**Published:** 1885-03

**Authors:** 


### CONTRIBUTORS.

| | |
|---|---|
| Hooper Alexander, Esq. | Atlanta, Ga. |
| Dr. W. T. Baird | Albany, Texas. |
| Dr. Robert Battey | Rome, Ga. |
| Dr. W. S. Bullard | Columbus, Ga. |
| Dr. A. W. Calhoun | Atlanta, Ga. |
| Dr. H. F. Campbell | Augusta, Ga. |
| Dr. R. O. Cotter | Macon, Ga. |
| Dr. A. C. Davidson | Sharon, Ga. |
| Dr. Jno. D. S. Davis | Birmingham, Ala. |
| Dr. W. H. Doughty | Augusta, Ga. |
| Dr. W. H. Elliott | Savannah, Ga. |
| W. S. Elkin | Atlanta, Ga. |
| Dr. R. O. Engram | Montezuma. Ga. |
| Dr. A. H. Evans | Carson, Texas. |
| Dr. E. J. Eve | Augusta, Ga. |
| Dr. Eugene Foster | Augusta, Ga. |
| Dr. J. McF. Gaston | Atlanta, Ga. |
| Dr. James A. Gray | Atlanta, Ga. |
| Dr. T. G. Hardman | Harmony Grove, Ga. |
| Dr. V. O. Hardon | Atlanta, Ga. |
| Dr. J. B. Hawthorne, D. D. | Atlanta, Ga. |
| Dr. Jno. Hemans | Boston. |
| Dr. Chas. Hicks | Dublin, Ga. |
| Dr. T. M. Holmes | Rome, Ga. |
| Dr. R. P. Huger | Birmingham, Ala. |
| Dr. J. M. Hull | Augusta, Ga. |
| Dr. Harry Huzza | Atlanta, Ga. |
| Dr. E. W. Lane | Scarboro, Ga. |
| Dr. J. C. Le Hardy | Savannah, Ga. |
| Dr. V. D. Lockhart | Homer, Ga. |
| Dr. H. McHatton | Macon, Ga. |
| Dr. T. M. McIntosh | Thomasville, Ga. |
| Dr. H. V. M. Miller, LL. D. | Atlanta, Ga. |
| Dr. K. P. Moore | Macon, Ga. |
| Dr. A. C. Moreland | Atlanta, Ga. |
| Dr. T. H. Nichols | Atlanta, Ga. |
| Dr. G. A. Noble | Atlanta, Ga. |
| Dr. R. J. Nunn | Savannah, Ga. |
| Dr. M. H. O'Daniel | Milledgeville, Ga. |
| Dr. W. O'Daniel | Bullard, Ga. |
| Dr. E. H. Richardson | Cedartown, Ga. |
| Dr. J. W. Russey | Rising Fawn, Ga. |
| Dr. O. O. Simpson | Norcross, Ga. |
| Dr. B. H. Smith | Blackshear, Ga. |
| Dr. J. C. Solmon | Perry, Ga. |
| Dr. J. P. Stevens | Macon, Ga. |
| Dr. R. H. Taylor | Griffin, Ga. |
| Dr. P. H. Thompson | Bluffton, Ga. |
| Dr. A. Thornburg | Ringgold, Ga. |
| Dr. W. F. Westmoreland | Atlanta, Ga. |
| Dr. Henry Wile | Atlanta, Ga. |
| Dr. H. J. Williams | Macon, Ga. |
| Dr. J. D. Wilson | Atlanta, Ga. |
| Dr. T. J. Word | Atlanta, Ga. |

### OUR ADVERTISERS.

PEPTONIZED COD LIVER OIL AND MILK.—This comparatively new preparation of cod liver oil prepared by Reed & Carnrick, New York, is the most palatable preparation of the kind we know. We are prescribing it, and find that patients complain less of it and improve more rapidly under it than any preparation of cod liver oil we have ever used.

PARKE, DAVIS & CO., DETROIT.—Read the attractive advertisement of this reliable firm to be found opposite "Clinic Reports," in this issue of THE JOURNAL. They are the most extensive manufacturers of elegant pharmaceutical preparations and new remedies in America.

SURGICAL INSTRUMENTS.—See advertisement of C. W. Lane & Bro., New York, and write them for catalogue and price list.

BUFFALO LITHIA WATER.—Attention is called to the advertisement of this celebrated water to be found on the first page of THE JOURNAL. By referring to the advertisement it will be seen that it is indorsed by the most eminent physicians in America.

A. S. ALOE & CO., ST. LOUIS.—By reference to advertising page 34 it will be seen that this firm succeeds Aloe, Hernstein & Co. The new firm is prepared to furnish any instrument or surgical appliance at low prices. Write them for catalogue and price list and mention THE JOURNAL.

CHARLES O. TYNER, ATLANTA.—Mr. Tyner, the "Corner Druggist," is headquarters for Hayden's Viburnum Compound and Peptonized Cod Liver Oil and Milk, two preparations that are being extensively used in this city.

L. H. BRADFIELD, ATLANTA.—This gentleman is prepared to fill orders for anything physicians may need. Orders by mail will be promptly attended to. He is thoroughly reliable in every respect.

MAGNUS & HIGHTOWER, ATLANTA.—These gentlemen carry a very large stock of drugs, chemicals, paints, oils, surgical instruments, etc., and are prepared to offer special inducements to physicians buying in large lots. They will sell goods as cheap as they can be sold by first-class houses anywhere. Read their advertisement and write them for prices.

BATTLE & CO., ST. LOUIS.—This firm has a new advertisement in this issue. Of the preparation advertised, Dr. M. W. Brubaker, of Charleston, Ill., says: When in St. Louis, I procured a bottle of Papine; before reaching home I took a very severe cold with consequent coughing and decided mucous irritation of the lungs, with dry inflamed condition of the throat. After suffering much from irritating attacks of coughing, I thought of Papine, and took a couple of doses. The effect was immediate and most happy. The coughing quickly ceased, and the dry, harsh condition of the throat and soreness of the lungs soon passed away. The effect of the remedy was a most signal success, and since then I have obtained similar results in other like cases.

